# The accuracy of clinician predictions of survival in the Prognosis in Palliative care Study II (PiPS2): A prospective observational study

**DOI:** 10.1371/journal.pone.0267050

**Published:** 2022-04-14

**Authors:** Patrick C. Stone, Christina Chu, Chris Todd, Jane Griffiths, Anastasia Kalpakidou, Vaughan Keeley, Rumana Z. Omar, Victoria Vickerstaff

**Affiliations:** 1 Division of Psychiatry, Marie Curie Palliative Care Research Department, University College London (UCL), London, United Kingdom; 2 Faculty of Biology, School of Health Sciences, Medicine and Health, The University of Manchester, Manchester Academic Health Science Centre, Manchester, United Kingdom; 3 Manchester University NHS Foundation Trust, Manchester, United Kingdom; 4 Palliative Medicine Department, University Hospitals of Derby and Burton NHS Foundation Trust, Derby, United Kingdom; 5 Department of Statistical Science, University College London (UCL), London, United Kingdom; Yale University School of Medicine, UNITED STATES

## Abstract

**Background:**

Prognostic information is important for patients with cancer, their families, and clinicians. In practice, survival predictions are made by clinicians based on their experience, judgement, and intuition. Previous studies have reported that clinicians’ survival predictions are often inaccurate. This study reports a secondary analysis of data from the Prognosis in Palliative care Study II (PiPS2) to assess the accuracy of survival estimates made by doctors and nurses.

**Methods and findings:**

Adult patients (n = 1833) with incurable, locally advanced or metastatic cancer, recently referred to palliative care services (community teams, hospital teams, and inpatient palliative care units) were recruited. Doctors (n = 431) and nurses (n = 777) provided independent prognostic predictions and an agreed multi-professional prediction for each patient. Clinicians provided prognostic estimates in several formats including predictions about length of survival and probability of surviving to certain time points. There was a minimum follow up of three months or until death (whichever was sooner; maximum follow-up 783 days).

Agreed multi-professional predictions about whether patients would survive for days, weeks or months+ were accurate on 61.9% of occasions. The positive predictive value of clinicians’ predictions about imminent death (within one week) was 77% for doctors and 79% for nurses. The sensitivity of these predictions was low (37% and 35% respectively). Specific predictions about how many weeks patients would survive were not very accurate but showed good discrimination (patients estimated to survive for shorted periods had worse outcomes). The accuracy of clinicians’ probabilistic predictions (assessed using Brier’s scores) was consistently better than chance, improved with proximity to death and showed good discrimination between groups of patients with different survival outcomes.

**Conclusions:**

Using a variety of different approaches, this study found that clinicians predictions of survival show good discrimination and accuracy, regardless of whether the predictions are about how long or how likely patients are to survive. Accuracy improves with proximity to death. Although the positive predictive value of estimates of imminent death are relatively high, the sensitivity of such predictions is relatively low. Despite limitations, the clinical prediction of survival should remain the benchmark against which any innovations in prognostication are judged.

**Study registration:**

ISRCTN13688211. http://www.isrctn.com/ISRCTN13688211.

## Introduction

Patients with advanced cancer, their families and the healthcare staff looking after them often want to know how long they can expect to live [1–3]. Earlier in the course of the illness, prognosis is usually measured in terms of years and is largely determined by the stage of the disease, tumour characteristics and the extent to which disease-modifying treatments are available or tolerated. However, by the time patients have developed advanced, progressive, incurable cancer and are receiving palliative care, prognosis is usually measured in terms of days, weeks or months. In these circumstances there are fewer algorithmics approaches to prognosticating and in day-to-day practice most prognoses are provided by clinicians using their experience, judgement and intuition [4].

Previous studies have evaluated the accuracy of clinician predictions and, in general, authors have reported that such predictions are inaccurate and over-optimistic [5,6]. However, these studies have been very heterogeneous [7] and that has made it difficult to generalise results. Studies have had methodological limitations such as: small sample sizes of clinicians [8,9] or patients [10]; minimal description of the characteristics of prognosticating clinicians beyond their professional background [11,12]; or a lack of clarity about how clinician estimates were obtained [11,13]. There has also been heterogeneity with regards to how “accuracy” has been defined (what level of error has been deemed acceptable) [10,14–16].

The Prognosis in Palliative Care Study II (PiPS2) was a large multi-centre observational cohort study of prognostic tools and clinician predictions of survival. The primary aim of the PiPS2 study was to validate the PiPS prognostic models [17–19] and secondary aims were to validate other prognostic scores [20]. As part of the PiPS2 study detailed data about clinical predictions of survival were collected independently from doctors and nurses and an agreed multi-professional estimate of survival was obtained. The primary purpose of collecting data about clinician predictions was to act as a benchmark against which to judge the performance of the various prognostic scores rather than to evaluate the accuracy of clinician predictions *per se*.

This report is a secondary analysis of data from the PiPS2 study to determine the accuracy of temporal (how long?) and probabilistic (how likely?) estimates of survival obtained from a large sample of doctors and nurses, relating to over 1800 patients.

## Methods

This was a secondary analysis of the PiPS2 data set. Detailed descriptions of the methods used in PiPS2 are available elsewhere [18–20]. Recruitment occurred between August 2016 and April 2018, and patients were followed up for a minimum of three months. Ethical approval for the PiPS2 study was granted from the Yorkshire & the Humber—Leeds East Research Ethics Committee (reference number: 16/YH/0132).

### Patient population

Patients were recruited from three settings: community palliative care teams (including day hospice and palliative care outpatients), hospital palliative care teams, and inpatient palliative care units. Eligible patients were those over 18 years with locally advanced or metastatic incurable cancer recently referred to palliative care services. Both patients with and without capacity to consent were recruited.

### Study assessments

#### Patient and clinician characteristics

Demographic details of enrolled patients were recorded. National Health Service (NHS) number and date of birth were required to determine date of death from NHS Digital. Information on performance status and site of primary tumour were also collected.

Doctors and nurses providing survival predictions were asked about their age, gender, professional training, and years of specialist experience. However, clinician participants were not required to provide their name, date of birth or other unique identifier.

#### Prognostic predictions

Shortly after patients were enrolled in the study, the attending doctor and nurse independently estimated their prognosis (in several different formats). Clinicians were first asked to make a broad decision about whether patients were likely to survive for “days” (0–13 days), “weeks” (2–7 weeks), or “months+” (2 months or longer). Clinicians were then asked to estimate survival more precisely using one of several pre-defined categories (≤1 week; >1 to 2 weeks; >2 to 3 weeks; >3 to 4 weeks; >4 to 5 weeks; >5 to 6 weeks; >6 to 8 weeks; >8 to 10 weeks; >10 to 12 weeks; and >12 weeks). Finally, clinicians were asked to estimate the probability of the patient surviving to specific time points (1 day, 3 days, 7 days, 15 days, 30 days, and 60 days) where 100% probability meant that the clinician thought that the patient was certain to survive as long as the specified time period and 0% indicated they believed that the patient was certain to die within that timeframe.

An agreed multi-professional estimate of survival was created using the broader estimates of survival (i.e. days, weeks, or months). If the doctor’s and nurse’s estimates independently agreed, this was taken to represent the agreed multi-professional estimate of survival. When their predictions were discordant, the doctor and nurse discussed the case and reached a consensus.

For each prediction, doctors and nurses were asked about the length of their relationship with the patient (less than a week, less than a month, less than 3 months, more than 3 months, or never met), and also when they last assessed the patient (today, in the last 3 days, in the last week, in the last month, over a month ago, or never met).

#### Actual length of survival

Dates of death were obtained from NHS Digital three months after the end of study recruitment. Length of survival was calculated from the date patients consented to participate until the date they died or date censored.

### Statistical methods

The database was checked for accuracy and missing values. This analysis used complete cases only (clinician prediction of survival and survival status up to two months [56 days]) no data were imputed. Clinician characteristics and prognostic predictions are reported using descriptive statistics. Categorical data are reported as numbers and percentages. Continuous variables are summarised with means and standard deviations or medians and interquartile (IQ) ranges.

For temporal estimates (i.e. about length of survival), accuracy was determined by concordance with actual length of survival. For probabilistic estimates, accuracy was assessed in several ways. Firstly, the observed proportion of patients who survived to each time point was compared to clinicians’ mean estimates of the probability of survival to that same time point. Secondly, Kaplan Meier graphs were used to summarise survival times of patients stratified according to the predictions made by clinicians. Finally, Brier’s scores [21] and the Index of Predictive Accuracy (IPA) [22] were computed for each probabilistic survival prediction. The Brier score ranges between 0 and 1. A score of 0 represents perfect accuracy and a score of 1 represents perfect inaccuracy.

The IPA has an advantage over the Brier’s score in that it is easier to see the added value of the model compared to a reference value for the event being predicted. To obtain the IPA measure, the Brier’s score is re-scaled. The value of IPA is calculated as 1 − (model Brier score/null model Brier score), where the null model contains no predictors. In the binary outcome setting where we are predicting if the patient is alive or not, the null model simply predicts the overall probability of survival at the specified time point in the study sample. IPA score ranges between negative infinity and 1. A score of 1 represents perfect accuracy and a score ≤ 0 is a useless model (i.e. the same or worse than the null model).

The doctors and nurses who provided survival estimates for patients in the study were not individually identified and clinicians sometimes provided survival estimates for more than one patient. In order to estimate the characteristics of individual clinicians, we assumed that people with the same age, gender, seniority and professional background working in the same institution were in fact the same individual.

## Results

A total of 1833 patient participants (with capacity to consent, n = 1610; without capacity to consent, n = 223) were recruited at 27 different sites in England and Wales. The mean age of patient participants was 70.2 years and 51.2% were men. The majority of patient participants were recruited from inpatient palliative care units. The most common tumour types were lung, head and neck and upper gastrointestinal cancers and the most common sites of metastases were bone, liver and lymph nodes. Most patient participants had a poor ECOG performance status, with 59.8% having a score of 3 or 4; 21.3% of patient participants were still receiving active oncological therapies. There was a minimum follow up of three months or until death (whichever was sooner; maximum follow-up 783 days). The median survival of patient participants was 45 days (IQR 16–140 days). The number of patient participants predicted to survive “days” was 405 (22.1%); “weeks” was 601 (32.8%); and months+ was 827 (45.1%).

It is estimated that the survival predictions were individually provided by 431 doctors and 777 nurses. Table 1 shows the characteristics of the clinicians and provides a summary of their familiarity with the patients about whom they were making prognostic estimates.

**Table 1:** Characteristics of doctors and nurses making predictions.

Characteristic	Doctors (n = 431)	Nurses (n = 777)
Gender, n (%) Male Female	117 (28.0) 301 (72.0)	36 (4.7) 732 (95.3)
Age (years), median (IQR)	35 (30, 44)	47.5 (37,54)
Specialty, n (%) Palliative care Other	360 (85.7) 64 (14.3)	755 (98.3) 13 (1.7)
Years since qualified, median (IQR)	9 (5, 20)	19 (9,30)
Years working in palliative care, median (IQR)	3 (0, 10.5)	6 (2, 12)
Length of relationship with patient, n (%)		
< 1 week	1211 (67.0)	1246 (68.3)
< 1 month	223 (12.3)	362 (19.9)
< 3 months	95 (5.3)	83 (4.6)
3 months+	38 (2.1)	47 (2.6)
Never met patient	241 (13.3)	86 (4.7)
Last assessed patient, n (%)		
Today	963 (53.2)	1295 (71.0)
Within last three days	409 (22.6)	211 (11.6)
Within last week	110 (6.1)	96 (5.3)
Within last month	72 (4.0)	118 (6.5)
Over one month ago	15 (0.8)	17 (0.9)
Never met patient	240 (13.3)	87 (4.8)

### Accuracy of temporal predictions

**Fig 1:**
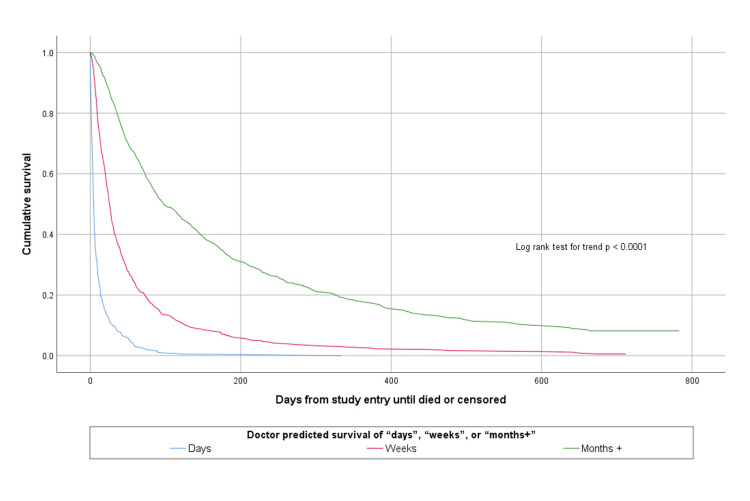
Kaplan Meier survival curves according to doctor estimated survival of days, weeks or months+.

**Fig 2:**
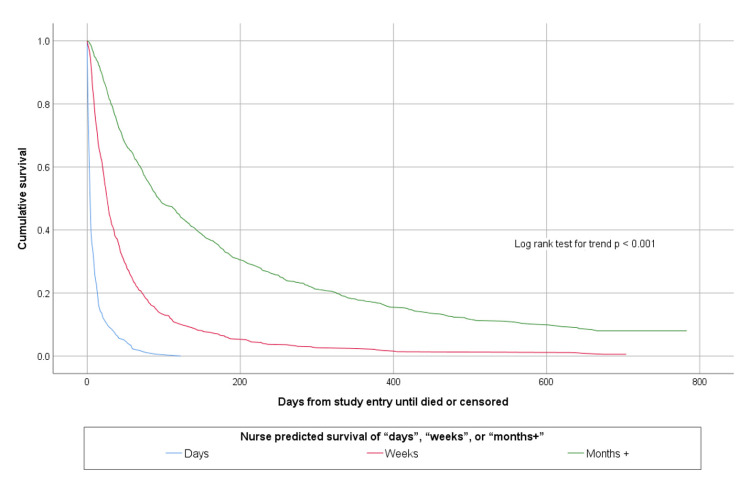
Kaplan Meier survival curves according to nurse estimated survival of days, weeks or months+.

The accuracy of agreed multi-professional estimates about whether patients would survive for days, weeks or months is shown in Table 2. The agreed multi-professional estimates were accurate on 1134/1831 (61.9%) occasions. Both doctors’ and nurses’ prognostic estimates were able to discriminate between patients with different survival prospects, as shown in Figs 1 and 2.

**Table 2:** Observed survival of patients compared to agreed multi-professional predictions.

Agreed multi-professional predictions	Observed survival			Total
	Days	Weeks	Months+	
Days	154	28	6	188
Weeks	215	321	160	696
Months+	36	252	659	947
**Total**	405	601	825	1831

**Fig 3:**
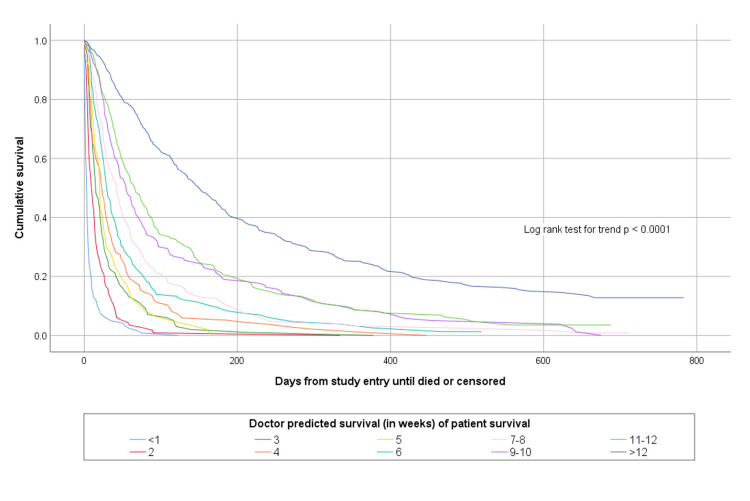
Kaplan Meier survival curves according to doctor estimated survival in weeks.

**Fig 4:**
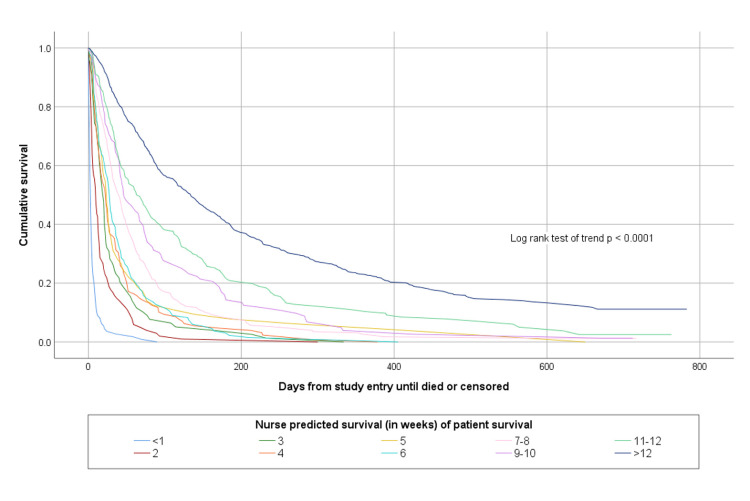
Kaplan Meier survival curves according to nurse estimated survival in weeks.

The accuracy of doctors’ and nurses’ more specific estimates about how long patients would survive is shown in Table 3. Overall doctors’ more specific temporal estimates were completely accurate on 591/1831 (32.3%) of occasions and nurses were completely accurate on 614/1831 (33.5%) of occasions. Both doctors’ and nurses’ more specific prognostic estimates were able to discriminate between patients with different survival prospects, as shown in Figs 3 and 4.

**Table 3:** Relationship between clinician predictions and actual survival.

Doctors	Observed survival (weeks)										
Estimated survival (weeks)	<1	2	3	4	5	6	7–8	9–10	11–12	>12	Total
Not given	9	6	1	2	0	0	1	2	1	2	24
< 1	94	13	6	1	1	1	2	2	1	1	122
2	49	26	12	8	3	9	1	2	1	3	114
3	23	23	11	15	5	2	6	2	5	7	99
4	25	27	10	18	10	7	10	4	4	20	135
5	6	11	10	4	3	2	3	2	2	4	47
6	11	27	17	21	16	10	14	8	6	29	159
7–8	12	20	25	24	11	12	25	17	12	50	208
9–10	5	8	9	18	13	9	17	11	11	52	153
11–12	6	10	18	11	11	18	23	16	16	89	218
>12	11	10	12	18	19	21	26	28	32	377	554
**Nurses**											
Not given	3	3	0	0	0	0	0	1	0	2	9
< 1	88	13	5	1	0	1	0	2	0	1	111
2	44	23	10	5	3	2	5	4	1	4	101
3	18	13	17	9	3	3	4	3	3	6	79
4	22	25	14	20	2	12	11	3	3	16	128
5	11	10	7	12	5	1	4	2	3	9	64
6	20	24	9	17	11	11	12	6	4	19	133
7–8	14	36	20	24	22	13	30	19	16	53	247
9–10	7	7	11	8	6	10	11	7	9	36	112
11–12	10	12	16	13	15	18	14	11	13	88	210
>12	14	15	22	31	25	20	37	36	39	400	639
Total	251	181	131	140	92	91	128	94	91	634	1833

### Accuracy of probabilistic predictions

**Fig 5:**
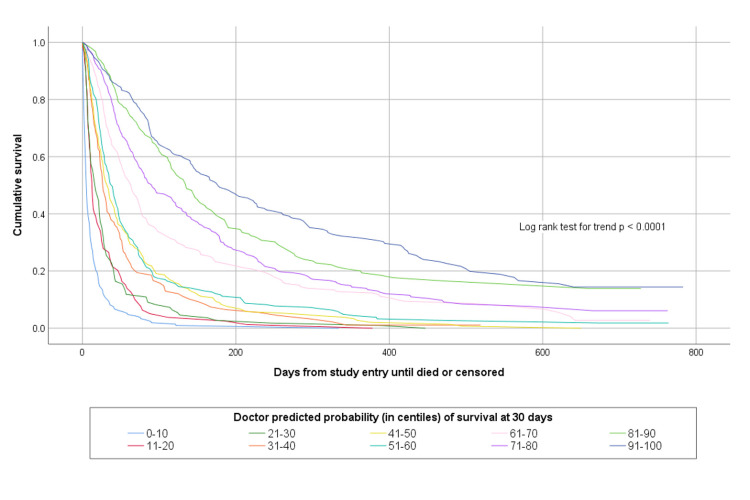
Kaplan Meier survival curves for patients according to doctor predicted probability of surviving 30-days.

**Fig 6:**
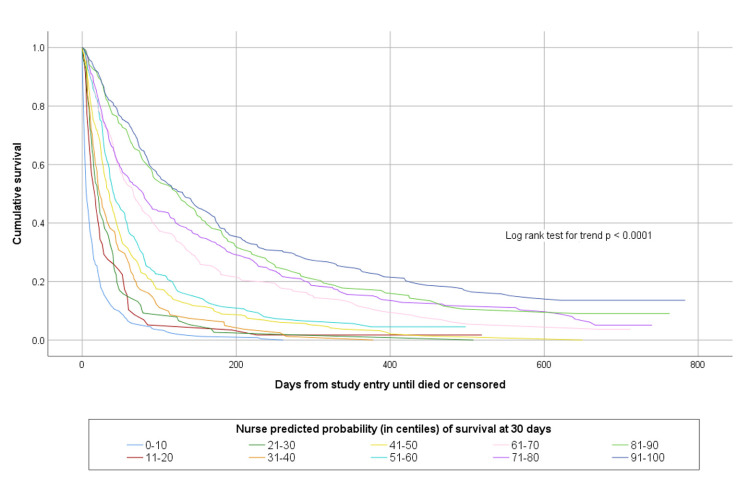
Kaplan Meier survival curves for patients according to nurse predicted probability of surviving 30-days.

Table 4 shows a comparison between doctors’ and nurses’ estimated probability of survival at various time points compared to observed survival. This shows that at a study sample level, the average survival predictions of clinicians were remarkably accurate. Clinicians’ probabilistic predictions were also able to discriminate between patients with different survival prospects. This is illustrated in Figs 5 and 6 which show the survival curves of patients according to their doctor- or nurse-predicted probability of surviving to 30-days, in deciles of incremental probability of survival.

**Table 4:** Comparison between predicted probabilities and observed survival.

Time point (days)	Doctors’ predicted mean probability of surviving (%)	Nurses’ predicted mean probability of surviving (%)	Observed survival (%)
**1**	95.5	96.4	97.5
**3**	90.6	91.6	94.1
**7**	83.2	84.5	86.3
**15**	73.1	74.1	75.0
**30**	58.2	59.8	60.3
**60**	43.1	45.0	43.2

Table 5 shows the Brier’s scores and IPA at each time point for doctors’ and nurses’ predictions. Both professional groups were better than chance at predicting survival to each time point, with doctors being consistently better than nurses. As expected, the performance of both groups was lower for longer time periods–an illustration of the so-called horizon effect. The performance of clinicians at predicting imminent death (i.e. within one week) was very good.

**Table 5:** Accuracy of doctor and nurse probabilistic predictions.

Clinician	Time point (days)	Brier’s score (95% confidence interval)^a^	IPA^b^
**Doctor**	**1**	0.016 (0.013, 0.020)	0.984
	**3**	0.036 (0.031, 0.042)	0.962
	**7**	0.07 (0.062, 0.078)	0.921
	**15**	0.129 (0.119, 0.139)	0.828
	**30**	0.169 (0.159, 0.178)	0.720
	**60**	0.183 (0.172, 0.193)	0.578
**Nurse**	**1**	0.017 (0.013, 0.022)	0.982
	**3**	0.04 (0.033, 0.046)	0.959
	**7**	0.071 (0.063, 0.08)	0.919
	**15**	0.143 (0.131, 0.154)	0.810
	**30**	0.192 (0.181, 0.204)	0.681
	**60**	0.201 (0.189, 0.212)	0.536

Notes

a For Brier’s scores lower is better.

b For IPA scores higher is better.

## Discussion

Clinicians in the PiPS2 study were good at positively identifying patients with a short prognosis. When doctors predicted that patients had less than one week left to live this prediction was correct on 94/122 occasions (Positive Predictive Value [PPV] = 77%). For nurses the equivalent figure was 79% (88/111). Moreover, if one takes into account the number of “near misses” (i.e. patients who were predicted to die within one week but actually died within two weeks) then the PPV for doctors increased to 88% (107/122) and the PPV for nurses increased to 86% (96/111). Where clinicians are less good is in identifying all of the patients who have a short prognosis. Thus, of the 251 patients who actually died within one week doctors only identified 94/251 (Sensitivity = 37%) of them, and nurses only identified 88/251 (Sensitivity = 35%).

It is more difficult for clinicians to predict precisely if, or when, a patient will die in subsequent weeks. Thus, for instance, doctors predicted that 99 patients would die between 2–3 weeks, but in fact only 11 patients died in that time frame. Therefore, these predictions were only completely accurate on 11/99 (= 11%) occasions. However, predicting death in such a narrow time frame is an artificial task and does not reflect real-world practice. Clinicians do not usually make a prediction about precisely when someone will die but rather the upper bound by which time death is expected. Therefore, it is probably more clinically meaningful to ask how accurate clinicians’ predictions are about predicting “death within three weeks” rather than their accuracy at predicting “death between two and three weeks”. Using this more clinically meaningful metric, the doctors in PiPS2 predicted that patients would die within three weeks on 335 occasions (122+114+99). In fact, patients died within three weeks on 226 occasions (94+49+26+11+23+23). Thus, the accuracy of doctors’ predictions about death within three weeks was 226/335 (= 67%). Moreover, if one includes the “near misses” (i.e. patients predicted to die within three weeks who actually died within four weeks) then the accuracy of doctor’s predictions increased to 250/335 (= 75%).

Although precise predictions concerning in which week a patient is expected to die are not clinically meaningful, there are occasions when placing both a lower (non-zero) and an upper bound on a prognostic estimate can be relevant. Thus, for instance, sometimes it is helpful to know that a patient is expected to live for more than “days” but less than “months”. This is a type and format of prognostic information that patients and their carers often prefer [18] and it accords with commonly used categories in clinical practice [23]. This type of prognosis can help with prioritising hospice admissions, assessing eligibility for certain benefits, arranging community care and helping families with planning end of life care. In the PiPS2 study, agreed multi-professional estimates of survival about whether patients would survive days, weeks or months+ were correct on 1134/1831 (= 62%) of occasions. For comparison, the “reference” baseline predictive accuracy for choosing between three categories is 33%.

It is relatively straightforward to determine the accuracy of temporal predictions of survival because they are generally either correct or incorrect binary outcomes (the patient is either dead or alive at the predicted time). However, even in this situation there is room for disagreement about the degree of latitude that should be allowed around a clinical prediction before it is considered inaccurate. It is, for instance, not necessarily the case that a prediction of three weeks’ survival should be judged to be inaccurate if the patient in fact dies after four weeks. Some authors have suggested that temporal predictions of survival should therefore be adjudged accurate if they fall in the range 67%-133% of actual survival [15,24,25]. Or maybe, for reasons discussed above, a more clinically meaningful range of accuracy could be defined as 0%-133% since patients who die earlier than expected still die within the upper bound of the prognostic estimate. However, determining (and interpreting) accuracy becomes more difficult when considering probabilistic rather than temporal predictions of survival. If a clinician predicts that a patient has a 60% chance of dying in the next four weeks, and they actually survive for longer than this, how does one determine if the prediction was or was not correct? In either eventuality the clinician could claim that their prediction was right. One way to conceptualise probabilities is that they refer to a “reference group” of similar patients. Thus a 40% probability of death within one month equates to a prediction that 40 out of 100 patients in a similar condition would die within one month. However, each patient is only one individual and they want to know what will happen to themselves rather than the fate of 99 other hypothetical similar patients.

In this study we evaluated probabilistic predictions in three ways. At a study sample level the probabilistic predictions were remarkably accurate. For example, across the sample of 1833 patients the average predicted probability of surviving for more than one day was 95.5% by doctors and 96.4% by nurses whereas the actual proportion of patients surviving for more than one day was 97.5%. Probabilistic predictions were similarly accurate across all of the evaluated timepoints. However, the interpretation of these findings is not straightforward. There were 1833 different patients, 431 doctors and 777 nurses involved in the study, and at most, each patient was assessed by two professionals. The results might illustrate the “wisdom of crowds” [26], but do not really say much useful about the value of probabilistic estimates for individual patients. When patients are grouped together according to their predicted probability of dying within a certain time frame, clinicians’ predictions are seen to have good discriminative ability. Patients with lower predicted probabilities of survival die sooner than patients with higher predicted probabilities of survival. Brier’s scores support the notion that clinicians’ predictions are accurate–that is, clinicians tend to attribute greater risk of death to patients who die than to those who survive.

In contrast to previous studies that have reported that clinician predictions of survival are inaccurate, we found that (when considered in clinically meaningful contexts), clinician’s predictions, particularly about imminent death, were good enough that they should remain the reference standard against which innovations in prognostication should be judged. Further work is required to investigate and understand how the accuracy of clinical predictions can be maximised. There is, for example, evidence that agreed multi-professional estimates are more accurate than uni-professional predictions [27], but there is no consensus about which disciplines should contribute to the prognostic decision, how many opinions are optimal or how differing viewpoints should be assimilated to reach a consensus on prognosis. There is some evidence that doctors who have known patients for longer, may be worse at prognosticating than doctors with a shorter relationship [15]. Many of the clinicians in our own study had only recently encountered the patients about whom they were providing prognostic estimates (67% of doctors and 68.3% of nurses had known the patient for <1week prior to making the prognosis). This may have contributed to the relatively good prognostic performance that we observed. Further work is also needed to understand the factors that contribute to clinical intuition. Which factors do expert clinicians focus on when formulating a prognosis? [7,28] There is a need to better understand what level of accuracy is clinically relevant for different groups. People understand that prognostication deals with uncertainty. It is likely however that patients, caregivers and professionals may have different expectations about the degree of error that is acceptable. Indeed, some patients may value the uncertainty that accompanies prognostic estimates because this can allow space for the fostering of realistic hope.
